# A Population-Based Study on Women Who Used Alcohol during Pregnancy and Their Neonates in Ontario, Canada

**DOI:** 10.3390/children11080993

**Published:** 2024-08-15

**Authors:** Svetlana Popova, Danijela Dozet, Valerie Temple, Catherine Riddell, Cathy Yang

**Affiliations:** 1 Institute for Mental Health Policy Research, Centre for Addiction and Mental Health, 33 Ursula Franklin Street, Toronto, ON M5S 2S1, Canada; danijela.dozet@camh.ca; 2 Institute of Medical Science, Faculty of Medicine, University of Toronto, Medical Sciences Building, 1 King’s College Circle, Toronto, ON M5S 1A8, Canada; 3 Dalla Lana School of Public Health, University of Toronto, 155 College Street, Toronto, ON M5T 3M7, Canada; 4 Factor-Inwentash Faculty of Social Work, University of Toronto, 246 Bloor Street W, Toronto, ON M5S 1V4, Canada; 5 Surrey Place, 2 Surrey Place, Toronto, ON M5S 2C2, Canada; valerie.temple@surreyplace.ca; 6 Better Outcomes Registry and Network (BORN) Ontario, 401 Smyth Road, Ottawa, ON K1H 8L1, Canada; criddell@childrenshealthcarecanada.ca (C.R.); cyang@bornontario.ca (C.Y.)

**Keywords:** prenatal alcohol exposure, prenatal substance exposure, neonatal abstinence syndrome, congenital anomalies, fetal alcohol spectrum disorder

## Abstract

Background: Data from birth registries can be studied to assess the prevalence of prenatal alcohol use and associated maternal and neonatal outcomes. Methods: Linked maternal and neonatal data (2015–2018) for alcohol-exposed pregnancies were obtained from the Better Outcomes Registry and Network (BORN) Ontario. Descriptive statistics were generated for maternal demographics, prenatal substance use, mental health/substance use history, and neonatal outcomes. Logistic regression models were performed to assess the odds of prenatal heavy (binge or weekly) alcohol and other substance use based on mental health/substance use history and other maternal demographics, and the impacts of heavy alcohol use and other prenatal substance exposures on neonatal outcomes. Results: A total of 10,172 (2.4%) women reported alcohol use during pregnancy. One-third had pre-existing or current mental health and/or substance use problems, which was associated with significantly higher odds of heavy alcohol use during pregnancy. Prenatal exposure to heavy alcohol use was associated with increased odds of neonatal abstinence syndrome (2.5 times); respiratory distress syndrome (2.3 times); neonatal intensive care unit (NICU) admission (58%); and hyperbilirubinemia (57%). Prenatal exposure to one or more substances in addition to alcohol was associated with significantly higher odds of fetal/maternal/placental pregnancy complications; preterm birth; NICU admission; low APGAR scores; one or more confirmed congenital anomalies at birth; respiratory distress syndrome; and intrauterine growth restriction. Conclusions: It is crucial to routinely screen childbearing-age and pregnant women for alcohol and other substance use as well as mental health problems in order to prevent adverse maternal and neonatal outcomes.

## 1. Introduction

Prenatal alcohol exposure remains an important public health concern globally, as women continue to consume alcohol and other substances during pregnancy, which is associated with many adverse maternal and neonatal outcomes [1,2,3]. Whereas the prevalence of tobacco use during pregnancy is slowly decreasing globally [4], there is evidence to suggest that alcohol during pregnancy may be increasing due to a variety of factors, including increased availability and accessibility of alcohol, alcohol marketing, and changes in attitudes towards the acceptability of women consuming alcohol in general [5,6].

One of the most serious outcomes associated with prenatal alcohol exposure is fetal alcohol spectrum disorder (FASD). This is a chronic neurodevelopmental disorder that affects an estimated 2–4% of the general population in Canada [7,8] and between 1 and 5% in the United States [9]. FASD is known to have physical, cognitive, and behavioral impacts that can lead to a wide variety of deficits and challenges [10]. Research has found a strong dose-dependent relationship between outcomes and exposure, whereby heavier maternal alcohol consumption and/or binge drinking lead to more deficits and challenges [11]. A recent meta-analysis of prospective longitudinal studies found significant and long-lasting effects on IQ, learning, memory, executive functioning and academic skills following prenatal alcohol exposure [12]. Even children with relatively lower levels of prenatal alcohol exposure are at risk of FASD [13] and problems with psychosocial functioning, sleep and adaptive functioning.

As compared to other substances, prenatal alcohol exposure can result in a broader range of adverse health outcomes across their lifespan, including more permanent comorbidities [14]. The harmful effects of prenatal alcohol exposure may be increased, however, if there is also exposure to additional substances [15], such as cocaine, cannabis, or opioids, which are known to negatively impact the child across their lifespan. Although research is more limited, available studies examining the effects of these substances on pregnancy outcomes have found that cannabis, cocaine and opioids are all associated with higher levels of behavioral challenges such as impulsivity, hyperactivity, and emotional reactivity [11]. Cognitive challenges such as executive functioning and attention deficits have also been reported in those prenatally exposed [11]. Prenatal exposure to substances in addition to alcohol is also associated with later adverse life experiences in children with FASD, such as substance misuse and involvement in the child welfare system [15]. Prenatal exposure to these additional substances can also complicate FASD assessment and diagnosis, for which there are already significant cost and capacity barriers [16,17,18]. This, in turn, may contribute to an increased cost burden for the healthcare system that is involved with diagnosis and support for individuals with FASD and prenatal alcohol exposure.

Numerous FASD prevention efforts are implemented concurrently in Canada, including pre-conception prevention education, education for women of childbearing age and their partners, provision of support to mothers with substance use issues, support to mothers with FASD, postpartum support for women and broad public awareness campaigns [19]. In addition, there is a national database that combines information regarding FASD diagnoses made in 26 clinics in provinces and territories across the country [20]. Despite these initiatives, Canada currently lacks a harmonized data collection system to gather information on children who have been prenatally alcohol- and/or substance-exposed [21], and the Public Health Agency of Canada has set forth a recommendation to increase national level surveillance in order to develop benchmarks for decision-making in policy [22].

Conducting surveillance on prenatal alcohol exposure by studying maternal characteristics associated with alcohol use in pregnancy and factors that can put women at-risk can provide important information for developing and implementing policies and programs for vulnerable individuals. For example, one Canadian study found that women with a history of substance use and mental health issues are more likely to have alcohol-exposed pregnancies [23]; however, Canadian women with alcohol-exposed pregnancies also tend to have delayed prenatal care, which can impede health education from prenatal care providers that is essential in preventing FASD in the child and reducing further teratogenic effects. In addition, attitudes toward alcohol use in general tend to predict alcohol use in pregnancy [24]. Together, this suggests that surveillance efforts should examine a range of maternal variables including mental health history, previous prenatal care, and substance use history. Gathering population-based information may be especially important for developing a broad and comprehensive view of the prevalence and effects of prenatal alcohol and substance exposure, particularly prenatal exposure to high levels (weekly or binge drinking) of alcohol and other substances in addition to alcohol, which can increase the teratogenic effects and negatively impact child development. Studies using databases focusing exclusively on individuals diagnosed with FASD may be subject to referral bias where those in particular sub-groups are more likely to be identified and sent for assessment than others. Population-based data, on the other hand, may be more inclusive of all those exposed.

The opportunity to study alcohol use during pregnancy and neonatal outcomes together is presented in exploring linked maternal–child records from birth registries. In the current study, maternal records with reported alcohol use in pregnancy and linked neonatal records were gathered from the provincial birth registry ‘Better Outcomes Registry & Network’ of Ontario, Canada for the fiscal years 2015–2018. The study sought information about the following: (1) the prevalence of self-reported alcohol use in pregnancy; (2) the maternal characteristics, mental health and substance use of women who consumed alcohol during pregnancy; (3) risk factors for heavy alcohol use during pregnancy; (4) the impacts of prenatal exposure to substances in addition to alcohol on neonatal outcomes; and (5) neonatal outcomes of alcohol-exposed pregnancies. To date, a study has not yet been conducted using linked maternal–neonate information in this setting.

## 2. Materials and Methods

### 2.1. Data Source

The Better Outcomes Registry & Network (BORN) Ontario is administered by the Children’s Hospital of Eastern Ontario (CHEO) and funded by the Ministry of Health and Long-Term Care (MOHLTC). BORN Ontario has captured information for all pregnancy, birth and neonatal outcomes in Ontario since 2012. The BORN Information System (BIS) collects data on all prenatal, intra-partum and post-partum encounters, including service utilization such as specialized antenatal screening, and neonatal intensive care unit (NICU) stay. The fiscal year of maternal and neonatal records (1 April–31 March, inclusive) is defined by the infant’s date of birth.

The BIS captures data on substance use during pregnancy, including exposure to alcohol, marijuana, cocaine, opioids, hallucinogens and gas/glue. These data are based on maternal self-reports of substance use to antenatal care providers during one or more antenatal encounters during the respective pregnancy, including the birth event. Alcohol use frequency is also reported (e.g., weekly or monthly), and when the frequency is unknown, the estimated total number of drinks is taken and averaged out over the entire antenatal period. At the birth event, neonatal data are collected on confirmed and suspected congenital anomalies at birth, including fetal alcohol syndrome (FAS) and other alcohol-related birth defects. Congenital anomalies, such as micrognathia and cleft palate, might indicate an alcohol-related birth defect (ARBD) [25].

A de-identified record-level dataset was obtained from BORN Ontario for mothers and neonates pertaining to alcohol-exposed pregnancies resulting in live births and stillbirths (born 1 April 2015 to 31 March 2018). Binary variables were created for each substance use, maternal mental health and neonatal outcome. To quantify heavy drinking during pregnancy, values were coded from the ‘alcohol use during pregnancy’ variable obtained in the dataset, which captured self-reported alcohol use frequency/quantity. A “yes” value in the ‘heavy drinking’ variable captured “episodic excessive drinking (binging)”, “more than one drink per week”, and “one drink per week”. All other values from the original alcohol use variable (“less than one drink per month”; “one drink per month”; “exposure, amount unknown”; and “exposure prior to pregnancy confirmed, amount unknown”) were coded as “no” in the new heavy drinking variable.

### 2.2. Data Analysis

To examine maternal characteristics and neonatal outcomes, descriptive statistics were generated using STATA 16.1 on all records containing data on the fiscal year, and these outcomes were stratified by fiscal year. A table with frequencies and percentages of suspected and confirmed congenital anomalies in neonatal records from this dataset were obtained separately from BORN Ontario, which did not contain information on fiscal year. Categories with counts below six were suppressed in the produced tables in order to protect patient privacy.

Logistic regression models were performed to achieve the following: (1) to explore the odds of heavy drinking (weekly or binge) and other substance use during pregnancy based on mental health/substance use history; (2) to explore the odds of adverse maternal/neonatal outcomes based on heavy drinking; and (3) to explore the odds of adverse neonatal outcomes based on prenatal substance exposure in addition to alcohol. Logistical regression models were performed on all maternal/neonatal records available, including those with missing data on fiscal year. The statistical significance of the odds ratios produced was set at the 0.05 alpha level.

## 3. Results

### 3.1. Prevalence

A record-level dataset with linked maternal and neonatal records was obtained for the alcohol-exposed pregnancies. There were a total of 10,308 neonates in the cohort in this time period. Data on fiscal year were missing for 395 neonatal records, and descriptive statistics generated for neonatal characteristics were restricted to records wherein fiscal year was available: a total of 9913 neonatal records. Using the pregnancy ID to eliminate duplicates (n = 136) from the linked dataset, it was found that there were 10,172 pregnant women in the dataset (i.e., over 100 multiple births in the cohort). Data on fiscal year were missing for 386 of these maternal records and descriptive statistics generated for maternal characteristics were restricted to records wherein fiscal year was available: a total of 9786 maternal records. Logistic regression models were run on all 10,172 women and the corresponding 10,308 neonates, as these analyses were not stratified by fiscal year.

In the 2015–2018 fiscal years, a total of 423,751 pregnancies in Ontario resulted in a live birth or stillbirth, of which 10,308 (2.4%) were alcohol-exposed, as captured in the BIS (Table 1).

### 3.2. Maternal Demographics

Age data were available in 10-year age brackets for the 9786 maternal records with data on fiscal year available. The majority (90.8%) of the pregnant women in this cohort were between the ages of 21 and 40 years (Table 2). These women were mostly nulliparous (52.6%) or primiparous (27.7%) and had zero or one biological children living at the time of the pregnancy (52.6%, and 27.8%, respectively).

### 3.3. Maternal Substance Use, Mental Health and Pregnancy Conditions

Forty-four percent of pregnant women in the cohort reported consuming an unknown amount of alcohol prior to pregnancy confirmation (Table 3). One third (32.9%) of women reported less than one drink per month during pregnancy, and 2.7% of women reported binge drinking. In total, 30.2% (n = 2937) of pregnant women in the cohort reported smoking at their first prenatal visit. A total of 18.5% (n = 1801) smoked less than 10 cigarettes per day, and 7.8% (n = 759) smoked 10–20 cigarettes per day. A total of 3055 women (31.2%) reported living with a smoker at the time of the first prenatal visit, indicating a possible risk of exposure to secondhand smoke.

Poly-substance use. As all women in the cohort had reported consuming alcohol during the respective pregnancy, additional exposures would indicate poly-substance exposure. A total of 1591 women (16.5%) reported drug and substance use during the respective pregnancy, which is defined as the use of street drugs or the inappropriate use of prescription or non-prescription drugs (Table 3). Notably, 928 pregnant women (9.7%) reported the use of marijuana; 133 women (1.4%) reported using cocaine; 265 (2.8%) reported using two drugs, and 82 (0.9%) reported using three or more drugs; these are all in addition to the self-reported use of alcohol during the respective pregnancy.

Mental Health issues. A total of 3219 pregnant women (33.3%) had one or more pre-existing or current mental health problems at the time of their pregnancy, which were either self-reported or diagnosed (Table 3). Anxiety (8.3%) was the most commonly self-reported/diagnosed mental health concern, followed by depression (5.9%) and addiction (1.8%). A reported 9.2% of pregnant women had two mental health concerns (excluding addiction). The majority (73.9%) of women in the cohort did not experience any fetal, maternal or placental pregnancy complications, and 77.8% had intended to exclusively breastfeed after childbirth.

### 3.4. Neonatal Outcomes

Among neonates in the cohort with available data on fiscal year (n = 9913), the majority were live births (99.6%) and were full-term at birth (between 39 and 41 weeks gestational age; 66.7%) or early-term (37–38 weeks gestational age; 24.4%) (Table 4). APGAR scores were in the normal range on average at the 1 min mark (mean score = 8.2) and the 5 min mark (mean score = 8.8). NICU admissions (Level 3) were fairly prevalent (15.2% overall) in this cohort. Though data on Level 3 NICU admissions are complete, BORN received additional patient data from only half of the Level 3 NICUs in Ontario during this time period, and information about neonates of past NICU admission is insufficient. For a variety of reasons, it is noted that neonatal health conditions for these years are very underreported, including neonatal abstinence syndrome (NAS). From the data available, however, it is indicated that 254 neonates (2.6%) in this cohort had a complication at birth, and notably, the most prevalent neonatal health condition (3.0% overall) was NAS, which is most commonly linked to prenatal exposure to opioids or stimulants.

### 3.5. Associations between Heavy Drinking during Pregnancy, Maternal Characteristics and Maternal/Neonatal Outcomes

Odds ratios were produced for heavy drinking (i.e., binge or weekly drinking during pregnancy) based on various maternal characteristics (Table 5). Reporting smoking in some quantity was associated with 32% higher odds of reporting heavy drinking during pregnancy, while reporting heavy smoking (10 or more cigarettes per day) was associated with 2.3 times higher odds of reporting heavy alcohol use. There was also a significantly higher odds of heavy alcohol use during pregnancy associated with reporting additional substances used during pregnancy: 3.47 times higher with cocaine (*p* = 0.000); 2.65 times higher with opioid use (*p* = 0.000); 57% higher with marijuana (*p* = 0.000); 4.86 times higher if two drugs were reported during pregnancy (*p* = 0.000); and 5.16 times higher if three or more drugs were reported during pregnancy (*p* = 0.000). Current or pre-existing anxiety reported in the maternal record seemed to be associated with 30% decreased odds of heavy alcohol use during pregnancy (*p* = 0.008). There was a higher odds of heavy alcohol use during pregnancy when addiction was reported in the maternal record: heavy alcohol use was 6.69 times higher if addiction was reported as the sole current/pre-existing mental health concern (*p* = 0.000); 6.07 times higher if reported as the mental health concern in addition to another mental health disorder (*p* = 0.000); and 3.55 times higher if reported in addition to two or more mental health disorders (*p* = 0.000). Associations between mental health concerns that excluded addiction and heavy alcohol use were not found to be clinically or statistically significant.

The odds of various adverse maternal/neonatal outcomes based on heavy alcohol use during pregnancy are displayed in Table 6. Heavy alcohol use during pregnancy was associated with a 41% higher odds of preterm birth (<37 weeks), even when adjusted for old maternal age (41 years and above) and polysubstance use during pregnancy (*p* = 0.001). Using adjusted models, heavy alcohol use during pregnancy was associated with significantly higher odds of the following: 2.51 times higher for neonatal abstinence syndrome (*p* = 0.000), 2.3 times higher for respiratory distress syndrome (*p* = 0.000), and 58% higher for NICU admission (*p* = 0.000). Adjusted models did not find clinically or statistically significant associations between heavy alcohol use during pregnancy and low APGAR scores at 1 or 5 min; the presence of one or more confirmed congenital anomalies at birth; intrauterine growth restriction; or any fetal/maternal/placental pregnancy complications. Heavy alcohol use was also associated with 57% higher odds of hyperbilirubinemia (*p* = 0.000).

Table 7 presents the odds of each adverse neonatal outcome based on prenatal substance exposures in addition to alcohol, irrespective of alcohol-use frequency or quantity during pregnancy. All of the odds estimates have been adjusted for maternal smoking status at the first prenatal visit (binary variable). The odds of any fetal/maternal/placental pregnancy complications were 59% higher if two drugs were used in addition to alcohol (*p* = 0.000) and by 18% if there was any substance used in addition to alcohol (i.e., any polysubstance use; *p* = 0.004). The odds of preterm birth (37 weeks) were 68% higher if there was prenatal exposure to marijuana (*p* = 0.000), by 2.47 times if there was prenatal exposure to two drugs in addition to alcohol (*p* = 0.000), and by 91% if there was prenatal exposure to any substance(s) in addition to alcohol (*p* = 0.000). The odds of admission to the NICU were higher for every substance reported to be used in addition to alcohol during pregnancy: 3.52 times higher for prenatal opioid exposure (*p* = 0.000); 83% higher for prenatal exposure to cocaine (*p* = 0.001); 40% higher for prenatal exposure to marijuana (*p* = 0.000); 3.83 times higher for prenatal exposure to two substances in addition to alcohol (*p* = 0.000); 7.91 times higher for prenatal exposure to three or more substances in addition to alcohol; and 2.58 times higher for prenatal exposure to any substance(s) in addition to alcohol (*p* = 0.000). The odds of hyberbilirubinemia were 37% higher for neonates prenatally exposed to marijuana (*p* = 0.010); 73% higher for neonates prenatally exposed to two or more drugs (*p* = 0.002); 96% higher for neonatal prenatally exposed to three or more drugs (*p* = 0.033); and 57% higher for neonates exposed to any drug(s) in addition to alcohol (*p* = 0.000). The odds of a low APGAR score at 1 min after birth (<7 score) were 40% higher for neonates prenatally exposed to marijuana (*p* = 0.001); 2.12 times higher for neonates prenatally exposed to three or more drugs in addition to alcohol (*p* = 0.006); and 40% higher for neonates prenatally exposure to any substance in addition to alcohol (*p* = 0.000). The odds of a low APGAR score at 5 min after birth (<7 score) were 39% higher for neonates with prenatal exposure to marijuana (*p* = 0.038); 1.97 times higher for neonates with prenatal exposure to two drugs in addition to alcohol (*p* = 0.002); and 71% higher for neonates with prenatal exposure to any substance in addition to alcohol (*p* = 0.000). There were 4.29 times higher odds of one or more confirmed congenital anomalies at birth for neonates with prenatal exposure to three or more substances in addition to alcohol (*p* = 0.001). The odds of respiratory distress syndrome were 3.30 times higher for neonates with prenatal exposure to two additional substances (*p* = 0.000) and 7.29 times higher for prenatal exposure to three or more additional substances (*p* = 0.000). For intrauterine growth restriction, the odds were 4.61 times higher for neonates with prenatal exposure to two additional substance(s) (*p* = 0.000).

When including neonatal records with missing values for fiscal year (n = 395) in the analysis, there were a total of 271 (2.6%) neonates with suspected or confirmed congenital anomalies at birth, out of the 10,308 neonates in the cohort. Notably, 21.0% of the data for the 271 neonates with suspected or confirmed anomalies are missing (Supplementary Table S1). A total of 175 neonates (1.7%) had one or more confirmed congenital anomalies at birth. The most prevalent confirmed congenital anomalies (among the 175 neonates) were cardiovascular: 20 neonates (7.4%) were diagnosed with an atrial septal defect (ASD); and 12 neonates (4.4%) were diagnosed with a ventricular septal defect (VSD). Ten neonates (3.7%) were confirmed to have malformations of the head and/or brain. Notably, there were no confirmed cases of FAS. The full list of confirmed congenital anomalies with corresponding frequencies and percentages is available in Supplementary Table S1.

## 4. Discussion

The prevalence estimate for self-reported alcohol use during pregnancy in an Ontario birth registry was found to be 2.4% in the current study, which is considerably lower than previous population-based estimates obtained using meta-analysis (approximately 10%) [26] and estimates gathered from phone health survey data (13.5%) [27]. While it may be that rates are lower in Ontario as compared to other jurisdictions, it is also the case that Canada lacks a harmonized approach to collecting information on alcohol use during pregnancy [21], and this may lead to differences in the frequency and extent to which prenatal care providers engage their pregnant patients in conversations about current alcohol and substance use. When asked about alcohol and other substance use during pregnancy, several factors may contribute to the under-reporting found in medical settings as compared to other settings, including stigma and shame, fear of judgment or consequences such as child apprehension, misunderstanding of purpose, and lack of trust in confidentiality. This presents a significant challenge for healthcare providers, researchers and policymakers charged with the deployment of resources to prevent alcohol use in pregnancy in a timely manner to at-risk populations of women.

Among mothers of live and stillbirths in Ontario who had reported alcohol use during pregnancy, 44.4% reported only consuming alcohol prior to pregnancy confirmation. This indicates that there may be some awareness of the risks of alcohol use during pregnancy among this population of women; however, this also highlights the need for prevention efforts aimed at encouraging abstaining from alcohol when trying to become pregnant or when pregnancy is suspected. Additionally, it underscores that unplanned pregnancies likely play a large role in the occurrence of alcohol use during pregnancy.

In this population of women with alcohol-exposed pregnancies, the majority (69.8%) of women did not smoke at the first prenatal visit or live with a smoker at that time (68.8%); the majority (66.6%) did not have pre-existing or mental health concerns during the pregnancy (comparable to the general population of Canada), the majority (83.5%) did not use substances in addition to alcohol; and the majority (90.3%) did not engage in heavy alcohol use during pregnancy. Though this is based solely on pregnancies with maternal self-report of alcohol use, these findings suggest that all women in the general population can and do consume alcohol during pregnancy, and it is not limited to women with these frequently studied risk factors. This demonstrates the importance of aiming public health and information initiatives about the harms of alcohol in pregnancy in a broad range of individuals.

Certain risk factors, however, were associated with heavy alcohol use (binge or weekly drinking) during pregnancy among women in this cohort. Using tobacco, cannabis, cocaine, and opioids were all associated with significantly higher odds of heavy alcohol use in pregnancy. In addition, the odds of heavy alcohol use tended to be higher as the number of other drugs used increased, with up to 5.16 times higher odds of an alcohol exposed pregnancy when three drugs or more in addition to alcohol were consumed. Overall, addiction, as captured in the BIS, is associated with heavy alcohol use during pregnancy. Indeed, heavy alcohol use was over six times more likely if addiction was reported as the sole current/pre-existing mental health concern or as the mental health concern in addition to another mental health disorder. This demonstrates the importance of collecting a history of substance use for pregnant patients to help identify patients who may need access to addiction or harm reduction services. Reporting high-risk populations of women should always be performed with the utmost care, however, and should be informed by an anti-oppressive lens that takes into account the intergenerational trauma, racial/cultural discrimination, language barriers, access to education and impacts of addiction-related stigma that are often experienced by women with alcohol-exposed pregnancies. It is also important to recognize that data often do not capture the reason or context behind substance use, which can be multifaceted and complex. It would allow for a more comprehensive treatment and support and, thus, better outcomes for women and their children.

Neonatal outcome data in this study show that the majority of neonates from these alcohol-exposed pregnancies were livebirths, full-term, with normal APGAR scores and low prevalence rates of neonatal health conditions that are typically associated with prenatal substance exposure (e.g., confirmed congenital anomalies; 2.6%). These findings need to be interpreted with caution, however, because not all neurodevelopmental disorders and deficits can be identified within the neonatal period. There were no cases of FAS registered during this study period. This is not uncommon that FAS diagnosis is not made or recorded at birth unless there are extreme cases (e.g., severe growth restriction and known prenatal heavy alcohol use). The majority of FAS cases may be diagnosed later after birth; therefore, birth registries typically do not capture all FAS cases, which affect the accuracy of data. An estimated 1 in every 67 children prenatally exposed to alcohol will develop FAS [26], and 1 in every 13 will develop FASD [28], meaning that in this cohort (10,308), roughly 154 of the neonates may have FAS, and 793 may be diagnosed with FASD later in life. Children with FASD are at higher risk of physical and mental health comorbidities [15], which will further increase their need for support services. These children may benefit from being screened for FASD at a later date, and maintaining a record of their prenatal exposure will assist with accurate diagnosis at that time.

Heavy alcohol use (binge or weekly drinking) during pregnancy, however, was significantly associated with several adverse neonatal outcomes, which illustrates the teratogenic effects of alcohol. Prenatal alcohol exposure to heavy alcohol levels was associated with higher odds of preterm birth (<37 weeks) by 41% when adjusted for old maternal age and polysubstance use during pregnancy. When adjusted for older maternal age and polysubstance use, heavy alcohol use was associated with significantly higher odds of NICU admission, neonatal abstinence syndrome, respiratory distress syndrome and hyperbilirubinemia. This demonstrates that heavy prenatal alcohol exposure, irrespective of additional substances used, can result in significant neonatal health complications that require increased services and therefore increased cost to the healthcare system and risk to the child.

This study also examined the impacts of prenatal substances in addition to alcohol on neonatal outcomes. Cocaine exposure was associated with significantly higher odds of Neonatal Abstinence Syndrome and NICU admission. Cannabis exposure was associated with significantly higher odds of preterm birth, NICU admission, hyperbilirubinemia, and low APGAR scores at 1 min and 5 min intervals. Opioid exposure was associated with a significantly and substantially higher odds of neonatal abstinence syndrome and NICU admission. The use of two drugs during pregnancy was associated with significantly higher odds of almost all outcomes observed. In addition, the use of two substances in addition to alcohol was associated with higher odds of neonatal abstinence syndrome, intrauterine growth restriction, NICU admission, respiratory distress syndrome, low APGAR scores at 5 min, preterm birth, and hyperbilirubinemia. Even more profound effects were found when examining the effects of prenatal exposure to three substances in addition to alcohol, especially for the odds of NICU admission. Lastly, when examining poly-substance use as a binary variable (i.e., the use of one or more drugs in addition to alcohol during pregnancy), polysubstance use was significantly associated with all of the adverse neonatal impacts studied. Together, these results emphasize the importance of access to stigma-free addiction support services for pregnant women that contain treatment for addiction to a range of prescription and illicit substances.

Whereas the majority of women in this cohort did not consume additional substances, those that did gave birth to neonates who experienced significant adverse outcomes that may impact the child across their lifespan. It is clear that other substance use is associated with increased odds of heavy alcohol use during pregnancy, and this can complicate both clinical diagnostic work and research aimed at studying the effects of various substances on pregnancy outcomes [11]. In clinical work, it will be important for prevention programs to focus on a range of substances and to have the capacity for concurrent treatments for multiple substance use disorders. Addressing mental health and substance use during pregnancy requires a multifaceted approach that integrates medical, psychological, and social support systems, which may include psychoeducation, peer support groups, telehealth services, integrated care models, and access to stigma-free interventions such as perinatal health programs and substance-use disorder treatment services.

This study has several strengths, including the extraction of the cohort from a population-based registry which captures a range of maternal and neonatal information for linked records of all pregnancies and births in Ontario. In addition, this study used a large sample of alcohol-exposed pregnancies to conduct an analysis of risk factors for heavy prenatal alcohol exposure and adverse neonatal outcomes, from which several recommendations can be made in light of a nationally recognized need for increased surveillance. For example, this study displays the importance of screening pregnant women for alcohol and other substance use at every prenatal encounter, as part of mandatory fields in the system.

This study also has several limitations, such as its reliance on maternal self-report for substance use during pregnancy and the absence of other information such as meconium testing. Data on the prevalence of alcohol use during pregnancy in Ontario are dependent on the thoroughness of antenatal screening for alcohol-use behavior from antenatal care providers, which is also unknown in these data. Available information on the frequency/quantity of reported alcohol use during pregnancy does not take into account the alcohol type(s) consumed or the timing of the respective pregnancy confirmation. The timing of pregnancy confirmation may be affected by unplanned pregnancy or limited access to prenatal care for women in this cohort; however, information on the timing of the first antenatal visit is also unknown in these data. Data on prenatal exposures to additional substances also do not take into account the frequency or timing of drug use, and the “two drugs” and “three drugs” categories do not specify which substances were used. Lastly, data were not collected or reported on paternal alcohol use, which may affect neonatal outcomes and/or offspring development [29]. Certain neonatal health conditions, especially neonatal abstinence syndrome, are often underreported. Lastly, this study could not directly study individual confirmed congenital anomalies and associated risk factors due to low cell counts.

## 5. Conclusions

It is crucial to screen childbearing age and pregnant women who use alcohol for mental health problems and other substance use and to facilitate access to treatment services to prevent adverse maternal and neonatal outcomes. Routine screening and data collection on alcohol use in pregnancy are urgently needed on provincial, national and global levels.

## Figures and Tables

**Table 1:** Prevalence of live and stillbirths of women with self-reported alcohol use during pregnancy in Ontario, 2015/16–2017/18.

Fiscal Year	All Live Births and Stillbirths of Women with Self-Reported Alcohol Use during Pregnancy		Live and Stillbirths of Women with No Self-Reported Alcohol Use		Total Number of Live Births and Stillbirths in Ontario (BIS)	
	n	% (Row)	N	% (Row)	n	% (Row)
2015/2016	3419	2.4	137,470	97.6	140,889	100.0
2016/2017	3506	2.5	137,784	97.5	141,290	100.0
2017/2018	3383	2.4	138,189	97.6	141,572	100.0
**ALL YEARS COMBINED**	**10,308**	**2.4**	**413,443**	**97.6**	**423,751**	**100.0**

**Table 2:** Demographic characteristics of women with alcohol-exposed pregnancies in Ontario, 2015/16–2017/18 (n = 9786).

Maternal Characteristics	2015/16 n = 3240	2016/17 n = 3332	2017/18 n = 3214	All Years Combined N = 9786
**Maternal age (at time of birth)**				
Below or at 20	264 (8.5%)	220 (6.9%)	199 (6.5%)	683 (7.3%)
21–30 years	1434 (46.2%)	1445 (45.1%)	1373 (44.8%)	4252 (45.4%)
31–40 years	1350 (43.5%)	1470 (45.9%)	1431 (46.7%)	4251 (45.4%)
41 and above	57 (1.8%)	69 (2.2%)	62 (2.0%)	188 (2.0%)
**Parity**				
0	1667 (53.7%)	1668 (52.2%)	1586 (51.8%)	4921 (52.6%)
1	837 (27.0%)	903 (28.2%)	853 (27.8%)	2593 (27.7%)
2	370 (11.9%)	404 (12.6%)	374 (12.2%)	1148 (12.3%)
3	122 (3.9%)	129 (4.0%)	128 (4.2%)	379 (4.1%)
4 or more	106 (3.4%)	94 (2.9%)	123 (4.0%)	323 (3.5%)
**Number of living children at the time of pregnancy**				
0	1664 (53.5%)	1678 (52.3%)	1600 (52.0%)	4942 (52.6%)
1	842 (27.1%)	907 (28.3%)	865 (28.1%)	2614 (27.8%)
2	378 (12.2%)	401 (12.5%)	361 (11.7%)	1140 (12.1%)
3	123 (4.0%)	129 (4.0%)	134 (4.4%)	386 (4.1%)
4 or more	102 (3.3%)	91 (2.8%)	119 (3.9%)	312 (3.3%)
**Number of previous term births**				
0	1732 (55.7%)	1732 (54.1%)	1657 (53.9%)	5121 (54.6%)
1	814 (26.2%)	906 (28.3%)	847 (27.6%)	2567 (27.4%)
2	367 (11.8%)	373 (11.7%)	349 (11.4%)	1089 (11.6%)
3	107 (3.4%)	111 (3.5%)	123 (4.0%)	341 (3.6%)
4 or more	89 (2.9%)	79 (2.5%)	98 (3.2%)	266 (2.8%)

**Table 3:** Substance use, mental health and pregnancy conditions among women with alcohol-exposed pregnancies in Ontario, 2015/16–2017/18.

	2015/16 n = 3240	2016/17 n = 3332	2017/18 n = 3214	All Years Combined N = 9786
**Alcohol use during pregnancy**				
Episodic excessive drinking (binging)	89 (2.8%)	77 (2.3%)	98 (3.1%)	264 (2.7%)
2–3 drinks per month	212 (6.5%)	175 (5.3%)	177 (5.5%)	564 (5.8%)
1 drink per week	115 (3.6%)	147 (4.4%)	105 (3.3%)	367 (3.8%)
>1 drink per week	114 (3.5%)	96 (2.9%)	108 (3.4%)	318 (3.3%)
1 drink per month	184 (5.7%)	226 (6.8%)	208 (6.5%)	618 (6.3%)
<1 drink per month	1123 (34.7%)	1068 (32.1%)	1027 (32.0%)	3218 (32.9%)
Exposure prior to pregnancy confirmation, amount unknown	1391 (42.9%)	1507 (45.2%)	1450 (45.1%)	4348 (44.4%)
Exposure, amount unknown	12 (0.4%)	36 (1.1%)	41 (1.3%)	89 (0.9%)
**Smoking at first prenatal visit**				
>20 cigarettes per day	77 (2.4%)	68 (2.1%)	55 (1.7%)	200 (2.1%)
10–20 cigarettes per day	272 (8.5%)	258 (7.8%)	229 (7.2%)	759 (7.8%)
<10 cigarettes per day	602 (18.7%)	598 (18.1%)	601 (18.8%)	1801 (18.5%)
Amount unknown	47 (1.5%)	61 (1.8%)	69 (2.2%)	177 (1.8%)
None	2215 (68.9%)	2324 (70.2%)	2240 (70.1%)	6779 (69.8%)
**Mother resides with smoker at first prenatal visit—Yes (%)**	1008 (31.1%)	1052 (31.6%)	995 (31.0%)	3055 (31.2%)
**Drug and substance exposure during pregnancy**				
Marijuana	304 (9.5%)	321 (9.8%)	303 (9.7%)	928 (9.7%)
Cocaine	40 (1.3%)	46 (1.4%)	47 (1.5%)	133 (1.4%)
Other	34 (1.1%)	31 (1.0%)	26 (0.8%)	91 (1.0%)
Opioids (misuse)	25 (0.8%)	28 (0.9%)	29 (0.9%)	82 (0.9%)
Gas/Glue	S	S	S	6 (0.1%)
Hallucinogens	S	S	S	S
2 drugs	79 (2.5%)	93 (2.8%)	93 (3.0%)	265 (2.8%)
3 or more drugs	28 (0.9%)	22 (0.7%)	32 (1.0%)	82 (0.9%)
None	2688 (83.9%)	2728 (83.4%)	2609 (83.1%)	8025 (83.5%)
**Pre-existing or current mental health concern(s)**				
Anxiety	260 (8.1%)	265 (8.1%)	276 (8.8%)	801 (8.3%)
Depression	183 (5.7%)	218 (6.7%)	168 (5.4%)	569 (5.9%)
Addiction	62 (1.9%)	43 (1.3%)	65 (2.1%)	170 (1.8%)
History of post-partum depression	52 (1.6%)	63 (1.9%)	50 (1.6%)	165 (1.7%)
Other	30 (0.9%)	45 (1.4%)	43 (1.4%)	118 (1.2%)
Bipolar	17 (0.5%)	24 (0.7%)	17 (0.5%)	58 (0.6%)
Schizophrenia	S	S	S	8 (0.1%)
2 mental health concerns (one of which is addiction)	37 (1.2%)	24 (0.7%)	35 (1.1%)	96 (1.0%)
3 or more mental health concerns (one of which is addiction)	48 (1.5%)	53 (1.6%)	48 (1.5%)	149 (1.6%)
2 mental health concerns (excl. addiction)	271 (8.4%)	293 (9.0%)	325 (10.3%)	889 (9.2%)
3 or more mental health concerns (excl. addiction)	64 (2.0%)	63 (1.9%)	69 (2.2%)	196 (2.0%)
None	2192 (68.1%)	2178 (66.6%)	2042 (65.0%)	6412 (66.6%)
**Prenatal classes—Yes (%)**	800 (24.7%)	821 (24.6%)	748 (23.3%)	2369 (24.2%)
**Intention to breastfeed**				
Yes, intends to exclusively breastfeed	2430 (78.7%)	2508 (77.6%)	2379 (77.2%)	7317 (77.8%)
Yes, intends to combination feed (breast milk/substitute)	320 (10.4%)	310 (9.6%)	339 (11.0%)	969 (10.3%)
No, does not intend to breastfeed	283 (9.2%)	340 (10.5%)	301 (9.8%)	924 (9.8%)
Mother unsure	55 (1.8%)	73 (2.3%)	62 (2.0%)	190 (2.0%)
**Any fetal/maternal/placental pregnancy complication(s)**	781 (24.4%)	887 (26.9%)	853 (26.9%)	2521 (26.1%)
**Hypertension disorder in pregnancy**				
Preeclampsia ^a^	129 (4.0%)	146 (4.4%)	134 (4.2%)	409 (4.2%)
Gestational hypertension	53 (1.6%)	57 (1.7%)	41 (1.3%)	151 (1.5%)
Pre-existing Hypertension ^b^	8 (0.3%)	8 (0.2%)	13 (0.4%)	29 (0.3%)
None	3046 (94.1%)	3119 (93.7%)	3020 (94.1%)	9185 (94.0%)
**Referred to specialized antenatal screening due to ultrasound abnormality**	43 (1.3%)	35 (1.1%)	25 (0.8%)	103 (1.1%)

S = Suppressed due to cell size < 6. ^a^—Includes: HELLP syndrome, eclampsia, preeclampsia, preeclampsia requiring magnesium sulfate and pre-existing hypertension with superimposed preeclampsia. ^b^—Not including pre-existing hypertension with superimposed preeclampsia.

**Table 4:** Outcomes of prenatally alcohol-exposed live and stillbirths in Ontario, 2015/16–2017/18 (n = 9913).

Outcomes at Birth	2015/16 FY n = 3288	2016/17 FY n = 3378	2017/18 FY n = 3247	All Years Combined N = 9913
**Birth outcome**				
Live birth	3143 (99.7%)	3230 (99.5%)	3107 (99.6%)	9840 (99.6%)
Stillbirth	10 (0.3%)	15 (0.5%)	14 (0.5%)	39 (0.4%)
Neonatal death	S	10 (0.3%)	7 (0.2%)	S
**Gestational age at birth (weeks)**				
<25 weeks	9 (0.3%)	13 (0.4%)	13 (0.4%)	35 (0.4%)
Preterm 25–33 weeks	55 (1.7%)	72 (2.2%)	68 (2.2%)	195 (2.1%)
Late preterm: 34–36 weeks	189 (5.6%)	188 (5.8%)	187 (6.0%)	564 (5.9%)
Early term: 37–38 weeks	791 (24.9%)	759 (23.5%)	771 (24.9%)	2321 (24.4%)
Full term: 39–41 weeks	2119 (66.7%)	2178 (67.4%)	2041 (65.9%)	6338 (66.7%)
Post-term: 42 weeks or more	16 (0.5%)	21 (0.7%)	16 (0.5%)	53 (0.6%)
**Neonatal level of care (LOC) of birth hospital**				
Neonatal Level I	543 (17.6%)	530 (16.7%)	480 (15.8%)	1553 (16.7%)
Neonatal Level IIa	391 (12.7%)	433 (13.7%)	422 (13.9%)	1246 (13.4%)
Neonatal Level IIb	657 (21.3%)	675 (21.3%)	658 (21.7%)	1990 (21.4%)
Neonatal Level IIc	906 (29.3%)	856 (27.0%)	872 (28.7%)	2634 (28.4%)
Neonatal Level IIIa/IIIb	591 (19.1%)	672 (21.2%)	603 (19.9%)	1866 (20.1%)
**Admission to neonatal intensive care unit (NICU)**	493 (15.5%)	492 (15.1%)	462 (14.9%)	1447 (15.2%)
**Apgar score (0–10): 1 min—Mean (SD)**	8.3 (1.6)	8.2 (1.6)	8.2 (1.7)	8.2 (1.6)
**Apgar score (0–10): 5 min—Mean (SD)**	8.8 (1.0)	8.8 (1.0)	8.8 (1.1)	8.8 (1.1)
**Neonatal birth complication(s)—Yes**	70 (2.2%)	76 (2.3%)	108 (3.6)	254 (2.6%)
Neonatal health conditions *				
Hyperbilirubinemia	220 (7.6%)	210 (7.0%)	222 (7.1%)	652 (7.3%)
Neonatal abstinence syndrome (NAS)	110 (3.4%)	92 (2.8%)	89 (2.9%)	291 (3.0%)
Confirmed congenital anomaly (any)—1 or more	56 (1.7%)	48 (1.4%)	53 (1.6%)	157 (1.6%)
Respiratory distress syndrome	31 (1.0%)	38 (1.2%)	50 (1.6%)	119 (1.2%)
Intrauterine growth restriction (IUGR)	32 (1.0%)	16 (0.5%)	30 (1.0%)	78 (0.8%)
Meconium aspiration syndrome (MAS)	7 (0.2%)	6 (0.2%)	13 (0.4%)	26 (0.3%)
Confirmed seizures treated pharmacologically	S	S	S	9 (0.1%)
Sepsis—positive blood culture	7 (0.2%)	S	S	14 (0.2%)
Other health conditions/hypertonia	S	S	S	11 (0.1%)

S = Suppressed due to cell size < 6. * Neonatal health conditions are not mutually exclusive; neonates may have more than one of the listed health conditions.

**Table 5:** Odds ratios of prenatal exposure to binge or weekly alcohol use based on maternal characteristics (n = 10,308).

Exposure Variable	Odds of Binge/Weekly Drinking (95% CI)	*p*-Value
Young maternal age (below or at 20)	0.96 (0.75, 1.24)	0.767
Advanced maternal age (41 and above)	0.81 (0.50, 1.30)	0.377
Smoking at first prenatal visit	1.32 (1.16, 1.52)	0.000 *
Heavy smoking (10 or more cigarettes per day) at first prenatal visit	2.30 (1.93, 2.73)	0.000 *
Marijuana use during pregnancy	1.57 (1.29, 1.90)	0.000 *
Cocaine use during pregnancy	3.47 (2.39, 5.04)	0.000 *
Opioid use during pregnancy	2.65 (1.62, 4.32)	0.000 *
2 drugs used during pregnancy	4.86 (3.77, 6.27)	0.000 *
3 or more drugs used during pregnancy	5.16 (3.31, 8.04)	0.000 *
One or more drugs used during pregnancy (binary)	3.01 (2.62, 3.46)	0.000 *
Current/pre-existing anxiety	0.70 (0.53, 0.91)	0.008 *
Current/pre-existing depression	0.93 (0.70, 1.24)	0.629
Current/pre-existing addiction	6.69 (4.98, 8.98)	0.000 *
History of post-partum depression	0.85 (0.50, 1.46)	0.568
Addiction + 1 mental health disorder	6.07 (4.05, 9.08)	0.000 *
Addiction + 2 or more mental health disorders	3.55 (2.50, 5.03)	0.000 *
2 mental health concerns (excl. addiction)	1.10 (0.88, 1.37)	0.396
3 or more mental health concerns (excl. addiction)	1.02 (0.64, 1.60)	0.944

* denotes a statistically significant relationship at the 0.05 alpha level.

**Table 6:** Odds ratios of adverse maternal/neonatal outcomes based on prenatal exposure to binge or weekly alcohol use (n = 10,308).

Outcome Variable	OR (95% CI)	*p*-Value	OR ^a^ (95% CI)	*p*-Value	OR ^b^ (95% CI)	*p*-Value	OR ^c^ (95% CI)	*p*-Value
Any fetal/maternal/placental pregnancy complication(s)	1.20 (1.04, 1.38)	0.012 *	1.20 (1.04, 1.39)	0.011 *	1.16 (1.00, 1.34)	0.038 *	1.15 (1.00, 1.33)	0.051
Preterm birth (<37 weeks)	1.61 (1.32, 1.97)	0.000 *	1.62 (1.32, 1.97)	0.000 *	1.41 (1.15, 1.73)	0.001 *	1.41 (1.14, 1.72)	0.001 *
Admitted to NICU	1.93 (1.66, 2.25)	0.000 *	1.93 (1.66, 2.25)	0.000 *	1.59 (1.36, 1.87)	0.000 *	1.58 (1.35, 1.85)	0.000 *
Hyperbilirubinemia	1.73 (1.39, 2.15)	0.000 *	1.73 (1.39, 2.15)	0.000 *	1.58 (1.27, 1.98)	0.000 *	1.57 (1.26, 1.96)	0.000 *
Neonatal abstinence syndrome (NAS)	4.57 (3.58, 5.84)	0.000 *	4.56 (3.57, 5.83)	0.000 *	2.58 (1.98, 3.35)	0.000 *	2.51 (1.92, 3.27)	0.000 *
APGAR Score < 7 at 1 min	1.08 (0.87, 1.32)	0.484	1.07 (0.87, 1.33)	0.494	1.00 (0.80, 1.23)	0.970	1.00 (0.80, 1.23)	0.968
APGAR Score < 7 at 5 min	1.45 (1.05, 2.01)	0.024 *	1.45 (1.05, 2.01)	0.024 *	1.28 (0.92, 1.78)	0.142	1.27 (0.91, 1.77)	0.157
Confirmed congenital anomaly (any)—1 or more	1.18 (0.74, 1.88)	0.496	1.18 (0.74, 1.89)	0.489	1.08 (0.67, 1.75)	0.745	1.08 (0.67, 1.74)	0.753
Respiratory distress syndrome	2.88 (1.93, 4.30)	0.000 *	2.88 (1.92, 4.29)	0.000 *	2.32 (1.53, 3.51)	0.000 *	2.30 (1.52, 3.47)	0.000 *
Intrauterine growth restriction (IUGR)	1.84 (1.03, 3.28)	0.038 *	1.86 (1.04, 3.31)	0.036 *	1.52 (0.84, 2.74)	0.168	1.49 (0.82, 2.70)	0.185
Sepsis—positive blood culture	1.83 (0.53, 6.35)	0.338	1.84 (0.53, 6.38)	0.334	2.06 (0.59, 7.26)	0.259	1.99 (0.56, 7.01)	0.285

^a^—adjusted for old maternal age; ^b^—adjusted for old maternal age and polysubstance use; ^c^—adjusted for old maternal age, polysubstance use and maternal smoking. * denotes a statistically significant relationship at the 0.05 alpha level.

**Table 7:** Odds of adverse neonatal outcomes based on prenatal substance exposures in addition to alcohol, adjusted for maternal smoking (n = 10,308).

	Cocaine	Marijuana	Opioids	2 Drugs	3 or More Drugs	ANY Poly-Substance Use (One or More)
Outcome Variable	OR (95% CI); *p*-Value	OR (95% CI); *p*-Value	OR (95% CI); *p*-Value	OR (95% CI); *p*-Value	OR (95% CI); *p*-Value	OR (95% CI); *p*-Value
Any fetal/maternal/placental pregnancy complication(s)	1.37 (0.97, 1.95), *p* = 0.076	1.04 (0.90, 1.22), *p* = 0.523	1.27 (0.82, 1.97), *p* = 0.282	1.59 (1.25, 2.03), *p* = 0.000 *	1.29 (0.82, 2.03), *p* = 0.270	1.18 (1.06, 1.32), *p* = 0.004 *
Preterm birth (<37 weeks)	1.48 (0.90, 2.44), *p* = 0.120	1.68 (1.37, 2.05), *p* = 0.000 *	1.21 (0.62, 2.34), *p* = 0.574	2.47 (1.82, 3.36), *p* = 0.000 *	1.80 (1.00, 3.27), *p* = 0.052	1.91 (1.63, 2.23), *p* = 0.000 *
Admitted to NICU	1.83 (1.25, 2.68), *p* = 0.002 *	1.40 (1.19, 1.66), *p* = 0.000 *	3.52 (2.32, 5.34), *p* = 0.000 *	3.83 (3.00, 4.89), *p* = 0.000 *	7.91 (5.13, 12.20), *p* = 0.000 *	2.58 (2.28, 2.91), *p* = 0.000 *
Hyperbilirubinemia	0.86 (0.43, 1.69), *p* = 0.656	1.37 (1.08, 1.74), *p* = 0.010 *	1.86 (1.01, 3.44), *p* = 0.048 *	1.73 (1.19, 2.51), *p* = 0.004 *	1.96 (1.06, 3.62), *p* = 0.033 *	1.57 (1.31, 1.88), *p* = 0.000 *
Neonatal abstinence syndrome (NAS)	6.99 (4.48, 10.88), *p* = 0.000 *	1.30 (0.92, 1.82), *p* = 0.135	14.39 (9.08, 22.79), *p* = 0.000 *	15.82 (11.83, 21.16), *p* = 0.000 *	27.33 (17.45, 42.80), *p* = 0.000 *	18.71 (14.17, 24.69), *p* = 0.000 *
APGAR Score < 7 at 1 min	0.87 (0.49, 1.54), *p* = 0.628	1.40 (1.15, 1.71), *p* = 0.001 *	1.02 (0.53, 1.98), *p* = 0.946	1.21 (0.84, 1.74), *p* = 0.299	2.12 (1.24, 3.62), *p* = 0.006 *	1.40 (1.20, 1.63), *p* = 0.000 *
APGAR Score < 7 at 5 min	0.83 (0.30, 2.27), *p* = 0.724	1.39 (1.00, 1.95), *p* = 0.050	1.24 (0.46, 3.43), *p* = 0.665	1.97 (1.20, 3.23), *p* = 0.007 *	1.75 (0.70, 4.35), *p* = 0.230	1.71 (1.33, 2.20), *p* = 0.000 *
Confirmed congenital anomaly (any)—1 or more	1.64 (0.60, 4.50), *p* = 0.332	0.98 (0.58, 1.63), *p* = 0.926	1.21 (0.30, 4.98), *p* = 0.787	1.01 (0.41, 2.47), *p* = 0.990	4.29 (1.84, 9.99), *p* = 0.001 *	1.48 (1.04, 2.10), *p* = 0.030 *
Respiratory distress syndrome	2.06 (0.75, 566), *p* = 0.161	1.75 (1.09, 2.81), *p* = 0.020 *	0.74 (0.10, 5.36), *p* = 0.766	3.30 (1.79, 6.08), *p* = 0.000 *	7.29 (3.44, 15.46), *p* = 0.000 *	2.79 (1.96, 3.97), *p* = 0.000 *
Intrauterine growth restriction (IUGR)	N/A	1.91 (1.07, 3.41), *p* = 0.028 *	1.20 (0.17, 8.78), *p* = 0.854	4.61 (2.34, 9.08), *p* = 0.000 *	2.62 (0.63, 10.86), *p* = 0.185	2.34 (1.48, 3.69), *p* = 0.000 *

* denotes a statistically significant relationship at the 0.05 alpha level.

## Data Availability

Restrictions apply to the availability of these data. Data were obtained from the BORN Ontario and are available from Dr. Svetlana Popova with the permission of the BORN Ontario and the Centre for Addiction and Mental Health.

## Supplementary Materials

The following supporting information can be downloaded at: https://www.mdpi.com/article/10.3390/children11080993/s1, Table S1: Frequency and percentage of confirmed congenital anomalies among prenatally alcohol-exposed neonates born in Ontario, 1 April 2015 to 31 March 2018 (n = 10,308).
