# Supplementary material for: A Population-Based Study on Women Who Used Alcohol during Pregnancy and Their Neonates in Ontario, Canada
Source: Children (Basel). 2024 Aug 15;11(8):993. doi: 10.3390/children11080993 (PMC11352585; doi:10.3390/children11080993)
Supplement: Supplementary file 1 [file children-11-00993-s001.zip › children-3140784-supplementary.pdf]

Table S1. Frequency and percentage of confirmed congenital anomalies among prenatally alcohol-exposed neonates born in Ontario, April 1, 2015 to March 31, 2018, (n=10,308)

Confirmed congenital anomaly	TOTAL	
	Number	Percentage (Column)
Missing Data	57	21.0
None (anomaly was suspected by not confirmed)	39	14.4
Cardiovascular \ Atrial septal defect (ASD)	20	7.4
Cardiovascular \ Ventricular septal defect (VSD)	12	4.4
Head-Cranium & Brain \ Other - malformations of the head & Brain	10	3.7
Extremities-skeletal \ Hands/feet-Club foot	9	3.3
Genitourinary Tract \ Hypospadias	8	3.0
Cardiovascular \ Other heart abnormalities	7	2.6
Face \ MOUTH-Cleft lip & palate	7	2.6
Genitourinary Tract \ Hydronephrosis (>10 mm)	7	2.6
Twins \ Dichorionic (DC) twins	6	2.2
Abdominal Wall \ Gastroschisis	<6	S
Abdominal Wall \ Omphalocele (exomphalos)	<6	S
Face \ MOUTH-Cleft palate	<6	S
Cardiovascular \ Arrhythmia	<6	S
Cardiovascular \ Tetralogy of Fallot (TOF)	<6	S
Chromosomes \ Trisomy 21 (Down syndrome)	<6	S
Extremities-skeletal \ Generalized/other-Congenital malformations of spine & bony thorax	<6	S
Extremities-skeletal \ Hands/feet-Polydactyly (feet)	<6	S
Genitourinary Tract \ Renal agenesis	<6	S
Gastrointestinal \ Other - malformations of the gastro-intestinal tract	<6	S
Head-Cranium & Brain \ Anencephaly	<6	S
Thorax \ Diaphragmatic hernia - Congenital (CDH)	<6	S
Thorax \ Other - congenital malformations of lung	<6	S
Abdominal Wall \ Umbilical hernia	<6	S
Abdominal Wall \ Other - congenital malformations of abdominal wall	<6	S
Cardiovascular \ Atrioventricular septal defect (AVSD) (endocardial cushion defect)	<6	S
Cardiovascular \ Premature closure of atrial septum (PFO)	<6	S
Cardiovascular \ Pulmonary (valve) atresia	<6	S
Cardiovascular \ Supra ventricular tachycardia (SVT)	<6	S
Face \ Other - malformations of the face	<6	S
Genitourinary Tract \ Other - malformations of urinary system	<6	S
Gastrointestinal \ Abnormal small bowel	<6	S
Gastrointestinal \ Duodenal atresia	<6	S
Head-Cranium & Brain \ Hydrocephalus	<6	S

Confirmed congenital anomaly	TOTAL	
	Number	Percentage (Column)
Spine - Back \ Other - malformations of the spine	<6	S
Structural-other \ Other - malformations not classified elsewhere	<6	S
Thorax \ Pulmonary hypoplasia	<6	S
Cardiovascular \ Ductus arteriosus - Patent (PDA)	<6	S
Cardiovascular \ Other - cardiac malformations not classified elsewhere	<6	S
Extremities-skeletal \ Generalized/other-Hip dislocation – congenital	<6	S
Extremities-skeletal \ Generalized/other-Skeletal dysplasia –other	<6	S
Extremities-skeletal \ Hands/feet-Adactyly (absent fingers/ toes)	<6	S
Extremities-skeletal \ Hands/feet-Clinodactyly (fifth finger)	<6	S
Extremities-skeletal \ Hands/feet-Polydactyly (hands)	<6	S
Extremities-skeletal \ Hands/feet-Syndactyly (feet)	<6	S
Extremities-skeletal \ Hands/feet-Syndactyly (hands)	<6	S
Face \ MOUTH-Cleft lip	<6	S
Genitourinary Tract \ Hydrocoele	<6	S
Genitourinary Tract \ Renal cyst	<6	S
Genitourinary Tract \ Undescended testicle(s)	<6	S
Genitourinary Tract \ Other - malformations of male genitalia	<6	S
Gastrointestinal \ Abnormal esophagus	<6	S
Gastrointestinal \ Tracheo-esophageal fistula (TEF)	<6	S
Head-Cranium & Brain \ Arnold Chiari malformation	<6	S
Spine - Back \ NTD (neural tube defect) with hydrocephalus	<6	S
Gastrointestinal \ Bowel obstruction small or large intestine	<6	S
Cardiovascular \ Aortic valve stenosis	<6	S
Cardiovascular \ Cardiomegaly	<6	S
Cardiovascular \ Coarctation of aorta	<6	S
Cardiovascular \ Double inlet ventricle (DIV)	<6	S
Cardiovascular \ Double outlet ventricle (DOV)	<6	S
Cardiovascular \ Aortic arch - hypoplastic	<6	S
Cardiovascular \ Hypoplastic left heart syndrome (HLHS)	<6	S
Cardiovascular \ Mitral valve dysplasia	<6	S
Cardiovascular \ Pericardial effusion	<6	S
Cardiovascular \ Pulmonary insufficiency	<6	S
Cardiovascular \ Single ventricle / univentricular connection	<6	S
Cardiovascular \ Transposition of great vessels (TGA)	<6	S
Chromosomes \ Trisomy 13	<6	S
Chromosomes \ Other	<6	S

Confirmed congenital anomaly	TOTAL	
	Number	Percentage (Column)
Congenital Infections \ Other - infections	<6	S
Extremities-skeletal \ Arms/legs-Fracture(s) - long bones	<6	S
Extremities-skeletal \ Generalized/other-Arthrogryposis multiplex congenital	<6	S
Extremities-skeletal \ Hands/feet	<6	S
Extremities-skeletal \ Hands/feet-Ectrodactyly (lobster-claw / cleft hand)	<6	S
Extremities-skeletal \ Hands/feet-Fused toes	<6	S
Extremities-skeletal \ Hands/feet-Webbed toes	<6	S
Extremities-skeletal \ Muscle/connective tissue disorders-Other - malformations of the musculoskeletal system	<6	S
Face \ EYES	<6	S
Face \ EYES-Hypertelorism	<6	S
Face \ MOUTH-Retrognathia	<6	S
Genitourinary Tract \ Bladder abnormalities	<6	S
Genitourinary Tract \ Cystic kidney(s) - other	<6	S
Genitourinary Tract \ Duplex kidney/collecting system	<6	S
Genitourinary Tract \ Ectopic/pelvic kidney	<6	S
Genitourinary Tract \ Multicystic kidney disease (MCKD)	<6	S
Gastrointestinal \ Atresia small or large intestine	<6	S
Gastrointestinal \ Hirschsprung's disease	<6	S
Gastrointestinal \ Imperforate anus	<6	S
Gastrointestinal \ Esophageal atresia	<6	S
Gastrointestinal \ Pyloric stenosis	<6	S
Head-Cranium & Brain \ Acrania	<6	S
Head-Cranium & Brain \ Dandy-Walker malformation / variant (DWM)	<6	S
Head-Cranium & Brain \ Holoprosencephaly	<6	S
Head-Cranium & Brain \ Macrocephaly	<6	S
Head-Cranium & Brain \ Megalencephaly	<6	S
Head-Cranium & Brain \ Ventriculomegaly - mild (10-15 mm)	<6	S
Neck \ Neck tumour – other	<6	S
Structural-other \ Amniotic Bands	<6	S
Syndromes \ Syndrome not otherwise specified	<6	S
Syndromes \ TAR (thrombocytopenia-absent radius) syndrome	<6	S
Thorax \ Lung cysts-other	<6	S
Thorax \ Pleural effusion(s) (hydrothorax)	<6	S
Structural-other \ Hydrops Fetalis	<6	S
**Total**	271	100.0

Categories are not mutually exclusive and, therefore, the percentages do not sum up to 100

S = Suppressed due to cell size <6.
